# The relationship of life satisfaction, worries, and media use: a population-based cross-sectional study in Germany

**DOI:** 10.1186/s12889-025-23818-6

**Published:** 2025-08-27

**Authors:** Lena-Marie Precht, Kathrin Schopf, Melanie Bunz, Ruth von Brachel, Lina Kinzenbach, Verena Pflug, Julia Brailovskaia, Christina Bartnick

**Affiliations:** 1 Mental Health Research and Treatment Center (FBZ), Ruhr University Bochum, Bochum, Germany; 2 Department of Child and Adolescent Psychology, Philipps University Marburg, Marburg, Germany; 3 German Center for Mental Health (DZPG), partner site Bochum-Marburg, Bochum, Germany

**Keywords:** Positive mental health, Worries, Social media, Media use, Crises, Dual-Factor Model

## Abstract

**Background:**

Socio-political challenges, climate crisis, wars and pandemics can significantly impact civilian mental health, leading to psychological impairments and decreased life satisfaction. We aimed to investigate the psychological factors and sources of information used by the German population after the COVID-19 pandemic, during a period marked by several ongoing crises.

**Methods:**

Overall, *N* = 1,008 individuals (51.2% female) aged 14 to 99 years (mean age 49.71 years) in Germany participated in the present population-based cross-sectional study. Two components of mental health were assessed using computer-assisted telephone interviewing: life satisfaction as a positive component and worries as a negative component. Additionally, bivariate and multivariate associations of media usage and these two variables were examined.

**Results:**

The majority of this representative sample (74.9%) reported experiencing life satisfaction often or very often, even though half of the sample (50.3%) also experienced frequent worries. Significant effects on life satisfaction were observed in relation to gender, education, and source of information: higher life satisfaction was reported by females, individuals with more years of education, and those who primarily relied on print media or radio for information. Furthermore, the results revealed significant associations between worries and regional factors as well as media usage: higher levels of worry were reported by individuals living in the states formerly considered East Germany, and by those who primarily used social media as their main source of information.

**Conclusion:**

This study provides first insights into life satisfaction and worries of the German population during ongoing crises one year after the end of the COVID-19 pandemic. The findings highlight the role of gender, education, region, and especially media usage in shaping the positive and negative aspects of mental health and underline the need for enhanced media literacy and targeted psychological interventions for vulnerable groups.

## Background

Over the past five years, global challenges such as the COVID-19 pandemic, climate and environmental crises, and political conflicts, including the wars in Ukraine and the Middle East, have created significant social and psychological stressors. These conflicts and crises pose a fundamental threat to human health and contribute to an increase in mental health problems [1, 2]. Research indicates that exposure to political violence can lead to various mental health conditions, ranging from acute stress reactions to long-term psychological disorders [2, 3]. For example, the ongoing conflict in Ukraine has displaced millions of civilians, triggering psychological stress linked to trauma and adversities that may contribute to mental health problems like post-traumatic stress disorder, depression or anxiety that are found in refugee populations [4]. Considering climate change and other environmental crises, the complex interplay of various factors can impact civilian mental health, leading to psychological impairments and functional disabilities [5, 6], and increasing the risk of developing mental disorders [6, 7]. For instance, the COVID-19 pandemic and its social consequences have emerged as major stressors, contributing to a decline in mental health, particularly among individuals with pre-existing mental health conditions [8–11] as well as among youths [12, 13]. In Germany, during and shortly after the COVID-19 pandemic, decreased life satisfaction and increased mental health problems were observed [14, 15]. Additional studies indicate that worries and fear of war are also associated with mental health issues in the population [15–17].

In the context of crises, media use plays a particularly important role. The type of media used to stay informed during crises can impact mental health and is associated with life satisfaction and mood [18–20]. People tend to consume more media during crises, especially if they have crisis-specific concerns [21, 22]. In a large Europe-wide panel survey, van Aelst et al. [22] found that during the COVID-19 pandemic, people more frequently used easily accessible media, such as television and social media, rather than printed media. Previous studies have shown that the intensity of social media use is linked to reduced well-being and mental health [18–20, 23]. During the COVID-19 pandemic, a positive relationship between social media use and increased burden and anxiety has been observed [24–26]. A critical issue pertaining to information presented on social media is that it is often characterized by an absence of balance and the presence of inaccuracies [27, 28]. This makes it difficult to distinguish opinions from facts. Therefore, fake news can be easily spread [28, 29]. Another concern is the growing influence of algorithms. Social media platforms use algorithms to shape users’ perceptions by displaying content that aligns with their existing preferences, reinforcing pre-existing beliefs, and making information appear even more imbalanced [27]. Furthermore, content creators on social media are not subject to the same regulations as journalists, such as those outlined in the German Press Code [30]. The Press Code requires that information must be truthful, respect human dignity, and undergo careful verification. However, it must be noted that the Press Code serves as a voluntary ethical framework for journalism [30] and unbalanced information or even misinformation might also appear in other media than social media, e.g., websites on the Internet, print media, television, or radio.

Our present study aims to examine the current mental health state of the German population one year after the official end of the COVID-19 pandemic [31], and during ongoing climate and environmental crises and political conflicts, including the wars in Ukraine and the Middle East. The Dual-Factor Model of mental health posits that mental health is not merely the absence of mental illness but consists of both positive and negative aspects (such as life satisfaction and worries about negative consequences) that coexist [32]. Based on this, we assess two components of mental health: life satisfaction as a positive component and worries as a negative component. Moreover, owing to the absence of prior studies that have examined this subject in the context of contemporary challenging times, we will exploratively examine the association between media usage and these two variables.

## Methods

### Procedure and participants

Data acquisition was performed by VERIAN, formerly KANTAR public, and took place in January 2024. Data were assessed using computer-assisted multi-topic telephone surveys (CATI = computer-assisted telephone interviewing) and conducted using state-of-the-art methods, which means that a mobile phone sample is used in addition to the landline sample. The combination of landline and mobile phone samples means that almost 100% of the population can be contacted. Although the use of this so-called dual-frame approach (ADM dual-frame design) is more complex, it optimizes the representativeness of the sample and provides for the use of 65% generated landline numbers and 35% generated mobile phone numbers. The telephone numbers of the landline sample are generated using the “Random Last Two Digits (RL(2)D) method” based on the so-called Gabler/Häder method [33]. Within the households, the persons to be interviewed are selected systematically using a random key. The selection basis was the ADM master sample for generated telephone numbers. The mobile phone sample is drawn in the same way, but the regional stratification is omitted here as no regional information is available from the ADM mobile phone sample. As mobile phones are used almost exclusively by one person, the systematic selection of target persons is also omitted for the mobile phone sample.

An iterative weighting of the collected data in several steps (equalization of the different selection probabilities of the individual target persons by design weighting and subsequent adjustment of the sample with regard to various characteristics to the demographic structures of the population taken from the current official statistics by means of redressement weighting) ensures that the sample on which the evaluation is based corresponds to the structure of the population in terms of its composition. This means that the survey results are representative and can be generalized to the population within the statistical error tolerances. Participants were recruited from all federal states in Germany aged 14 years and above. Participation was not compensated. The local Ethics Committee of the Ruhr-University Bochum approved the implementation of the study. The overall sample is comprised of 1.008 participants (51% women). Demographics are presented in Table 1.

**Table 1:** Demographic characteristics of the sample

	M (SD)	%	Range (age in years)
Sex: *female*		51.2	
Region of residence: *west*		82.8	
Level of education:			
*Low level* ^*1*^		33.0	
*Medium level* ^*2*^		30.2	
*High level* ^*3*^		33.6	
Age	49.71 (20.04)		14–99
Age group:			
*Adolescent or young adult*		18.6	14–29
*Middle age*		55.4	30–64
*Regular retirement age*		26.0	> 65

^1^ 9–10 years of education, ^2^ 11–13 years of education, ^3^ 13 years of education including A-levels or college diploma

### Measures

#### Life satisfaction

The Positive Mental Health Scale (PMH-Scale) [34] is a unidimensional scale assessing the level of positive mental health. The PMH-Scale is an internationally well-established instrument for the assessment of psychological, emotional, and social well-being. The original PMH-Scale includes nine items that are rated on a 4-point Likert-type scale (e.g., “I enjoy my life”; 0 = do not agree, 3 = agree). In the current study, item No. 3 (“All in all, I am satisfied with my life”) was implemented to assess life satisfaction as a component of positive mental health. Response modalities were adapted to the same form as all other instruments (1 = *very rarely*, 2 = *rarely*, 3 = *sometimes*, 4 = *often*, 5 = *very often*).

#### Worries

As to our knowledge there is no validated item to measure the level of worries regarding political developments on one’s personal life or family, we used an author constructed scale. The level of worries was administered using the item: “How often are you currently afraid or worried that political developments in the world could have a negative impact on your life or the life of your family?”, rated on the same 5-point Likert-type scale as the above-mentioned instrument (1 = *very rarely*, 2 = *rarely*, 3 = *sometimes*, 4 = *often*, 5 = *very often*).

#### Media use

Media use was assessed using an author constructed scale: “How do you usually get informed about current events?”. The following categories were assessed in random order:

- Internet 
- Print media
- Television
- Social Media (e.g., WhatsApp, Facebook, YouTube, Instagram, TikTok, Pinterest or Twitter/X)
- Radio
- I don’t know, no answer

### Statistical analyses

Statistical analyses were conducted using SPSS 27 software. After descriptive statistics (means, standard deviations, frequencies), Pearson’s correlations and chi-squared tests with *r* and Cramér’s *V* as effect-size measures were calculated to examine the relationship between sociodemographic characteristics (i.e., age, age group, gender, region of residence, years of education) and the variables of interest (life satisfaction, worries about the negative impact of political developments on one’s own life, main source of information regarding daily news). In case of expected cell frequencies below five, exact chi-squared tests were applied. Subsequently, a one-way multivariate analysis of covariance (MANCOVA) and adjusted Bonferroni post-hoc tests were performed to test group differences in life satisfaction and worries as a function of gender, region of residence, years of education, and main source of information regarding daily news. Age was included as a covariate. Wilks’ Lambda (Λ) served as the test statistic and partial eta-squared (η^2^_p_) as the effect-size measure. All analyses were conducted at a two-tailed level of significance of *p* <.05. Due to the large sample size and the assumption that data were missing at random, missing data were deleted listwise [35].

## Results

### Descriptive characteristics of sample

A total of 1.008 participants completed the survey, with 51.2% being female and 48.8% male. On average, participants were 49.71 years old (*SD* = 20.04, range: 14–99). Nearly one-fifth of participants were adolescents or young adults (18.6%, ages 14–29), approximately half were middle-aged (55.4%, ages 30–64), and about one-fourth had reached the standard retirement age in Germany (26.0%, ages 65 and above). The large majority of participants (82.8%) reported living in states formerly part of West Germany, while 17.2% lived in states formerly part of East Germany. Approximately one-third of participants had a low level of education (33.0%, 9–10 years), a medium level (30.2%, 11–13 years), or a high level of education (33.6%, 13 years including A-levels or a college diploma). The descriptive characteristics of the sample are shown in Table 1. When compared to data from the Federal Statistical Office of Germany [36], the characteristics of the present sample closely resemble the population characteristics of Germany in terms of age, sex, and region of residence.

**Fig. 1:**
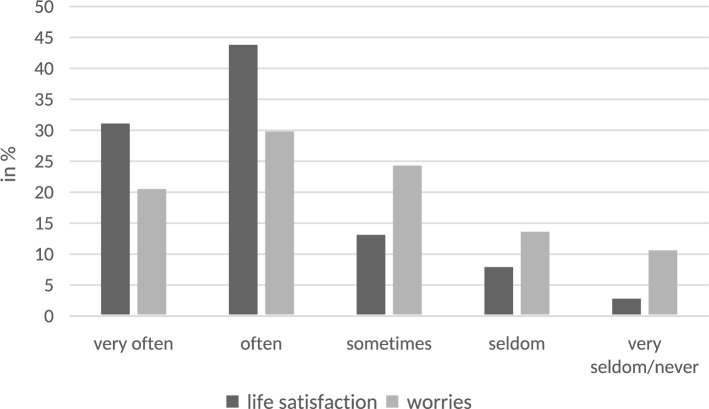
Overview of the sample’s unadjusted response pattern regarding mean life satisfaction and worries

Nearly three-quarters of the sample reported experiencing life satisfaction either very often (31.1%) or often (43.8%). Additionally, 13.1% reported experiencing it sometimes, 7.9% seldom, and 2.8% never or very seldom. For 1.2% of the sample, no data on life satisfaction was available. Worries about the negative impact of political developments on their own lives were commonly reported: 20.5% of the sample reported experiencing them very often, 29.8% often, 24.3% sometimes, and approximately one-quarter experienced them seldom (13.6%) or never/very seldom (10.6%; see Fig. 1 for the unadjusted response pattern). For 1.1% of the sample, no data on worries was available. Most participants reported that their main source of information for daily news was television (26.3%) or the Internet (24.3%), followed by print media (newspapers or magazines, 15.2%) and radio (16.3%). A total of 12.7% reported that social media (e.g., Facebook, YouTube, Instagram, TikTok, or X) was their primary source of daily news.

### Bivariate and multivariate associations between sociodemographic variables, life satisfaction, worries, and main source of information

There was a significant small negative association between worries about the negative impact of political developments on one’s own life and age (*r* = −.158, *p* <.01) as well as life satisfaction (*r* = −.096, *p* <.01), indicating that persons who experience worries more frequently than others tend to be younger and to experience somewhat lower life satisfaction. Although the correlation between age and life satisfaction was not significant (*r* =.033, *p* =.300), the chi-squared test between life satisfaction and age group (χ²(8) = 26.34, *p* <.001, *V* = 0.12) points to a small significant relationship between both variables. Except for the relationship between age and life satisfaction, age group and sex (χ²(2) = 1.43, *p* =.490, *V* = 0.04), life satisfaction and region of residence (χ²(4) = 8.48, *p* =.075, *V* = 0.09), sex and region of residence (χ²(1) = 0.17, *p* =.683, *V* = 0.01), and media use and region of residence (χ²(4) = 6.32, *p* =.176, *V* = 0.08) all relationships between the investigated variables were significant. Pearson’s correlations are presented in Table 2. The results of the chi-squared tests are presented in Table 3.

**Table 2:** Pearson’s correlations

Variables	Life satisfaction	Worries	Age
Life satisfaction	1		
Worries	− 0.096**	1	
Age	.033^ns^	− 0.158**	1

^ns^ non-significant, ** *p* <.01

**Table 3:** Pearson’s chi-squared tests

Variables	Life satisfaction	Worries	Sex	Age group	Region	Source
Sex	38.89***	12.30*				
Age group	26.34**	39.19***	1.43^ns^			
Region	8.48^ns^	16.52**	0.17^ns^	11.77**		
Source	59.92***	58.62***	9.67*	200.15***	6.32^ns^	
Education	49.73***	35.85***	7.32*	29.76***	21.24***	77.48***

^ns^ non-significant, *p <.05, **p <.01, ***p <.001; source = source of information regarding daily news

The MANCOVA (*N* = 831) showed a statistically significant difference between sex (*F*(2, 820) = 15.882, *p* <.001, η^2^_p_ = 0.037, Wilk’s Λ = 0.963), region of residence (*F*(2, 820) = 14.349, *p* <.001, η^2^_p_ = 0.034, Wilk’s Λ = 0.966), years of education (*F*(4, 1640) = 6.725, *p* <.001, η^2^_p_ = 0.016, Wilk’s Λ = 0.968), and media use (*F*(8, 1640) = 4.050, *p* <.001, η^2^_p_ = 0.019, Wilk’s Λ = 0.962) on the combined dependent variables life satisfaction and worries about the negative impact of political developments on one’s own life. The covariate age did not serve as a significant predictor of the dependent variables (*F*(2, 820) = 1.372, *p* =.254, η^2^_p_ = 0.003, Wilk’s Λ = 0.997). Adjusted Bonferroni post-hoc tests were conducted for both dependent variables (see Table 4). Life satisfaction was dependent on sex (*F*(1, 821) = 28.744, *p* <.001, η^2^_p_ = 0.034), years of education (*F*(2, 821) = 13.385, *p* <.001, η^2^_p_ = 0.032), and media use (*F*(1, 4) = 5.581, *p* <.001, η^2^_p_ = 0.026), but not dependent on region of residence (*F*(1, 821) = 1.810, *p* =.179, η^2^_p_ = 0.002). The experience of worries was dependent on region of residence (*F*(1, 821) = 28.024, *p* <.001, η^2^_p_ = 0.033) and media use (*F*(4, 821) = 2.650, *p* <.05, η^2^_p_ = 0.013), but not dependent on sex (*F*(1, 821) = 1.506, *p* =.220, η^2^_p_ = 0.002) or years of education (*F*(2, 821) = 0.227, *p* =.797, η^2^_p_ = 0.001).

**Table 4:** Adjusted Bonferroni post-hoc tests for both dependent variables

Dependent Variable		*F*	Sig.	η^2^_*p*_
Life satisfaction	Sex	28.744	0.000	0.034
	Region of residence	1.810	0.179	0.002
	Education	13.385	0.000	0.032
	Source of information	5.581	0.000	0.026
Worries	Sex	1.506	0.220	0.002
	Region of residence	28.024	0.000	0.033
	Education	0.227	0.797	0.001
	Source of information	2.650	0.032	0.013

Age was included as a covariate in the MANCOVA

**Fig. 2:**
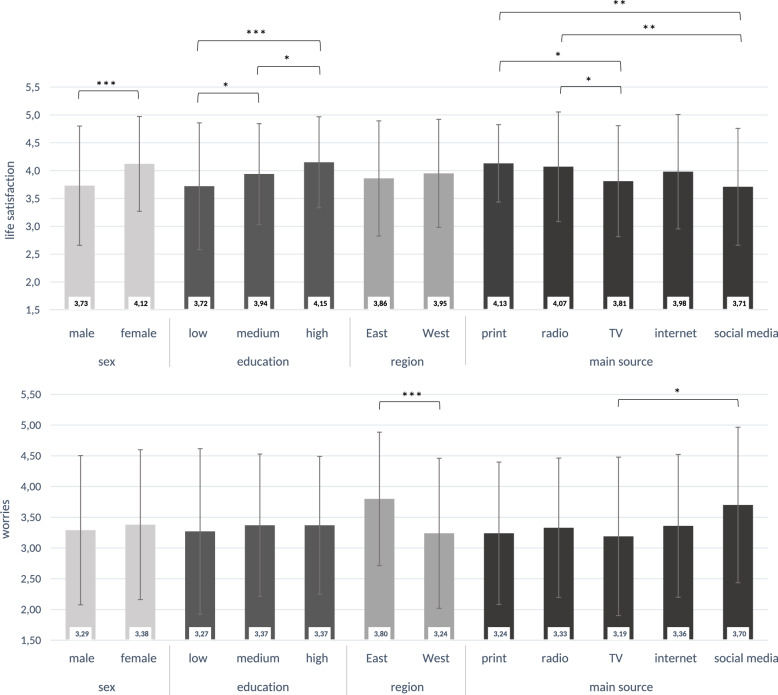
Mean level of life satisfaction and worries

When considering life satisfaction, females reported significantly higher satisfaction than males (*M* = 4.12, *SD* = 0.85 vs. *M* = 3.73, *SD* = 1.07). Individuals with A-levels or college diplomas reported higher life satisfaction (*M* = 4.15, *SD* = 0.82) compared to those with 11–13 years of education (*M* = 3.94, *SD* = 0.90), who, in turn, reported higher satisfaction than those with 9–10 years of education (*M* = 3.72, *SD* = 1.14). People who primarily used print media (newspapers or magazines) reported significantly higher life satisfaction (*M* = 4.13, *SD* = 0.70) than those who used television (*M* = 3.81, *SD* = 1.00) or social media (*M* = 3.71, *SD* = 1.05). Similarly, radio use was associated with significantly higher life satisfaction (*M* = 4.07, *SD* = 0.98) compared to television or social media use. In terms of worries, people who primarily used television reported fewer worries than those who primarily used social media (*M* = 3.19, *SD* = 1.29 vs. *M* = 3.70, *SD* = 1.27). Additionally, individuals living in states formerly considered part of West Germany reported significantly lower worries than those in states formerly considered East Germany (*M* = 3.24, *SD* = 1.22 vs. *M* = 3.80, *SD* = 1.09), as shown in Fig. 2.

## Discussion

The primary goal of our study was to illuminate the current mental health state of people in Germany, particularly considering the politically challenging times. To the best of our knowledge, no comparable study has considered the current political, environmental, and climate crises post-COVID-19 in Germany, and their connection to life satisfaction and worries.

We assessed mental health through two components: life satisfaction as a positive component and worries as a negative component. Additionally, we explored the potential relationship between these variables and the role of media use in this context. We conducted a population-based cross-sectional study involving 1,008 participants aged 14 to 99 years in Germany. The main results of this study indicate that the majority of the representative sample experiences life satisfaction often or very often, even though half of the sample also experiences frequent worries. A significant gender effect was found: females reported higher levels of life satisfaction than males. However, both female and male participants reported similar levels of worry. Furthermore, a significant regional effect was observed, with participants from East Germany reporting more worries than those from West Germany, although no differences in life satisfaction were found between the two regions. Additionally, we found a significant effect of education, where life satisfaction increased with higher levels of education, while worries remained unchanged. Finally, the most frequently used sources of information were television and the Internet. However, the use of print media was significantly correlated with higher life satisfaction than the use of television or social media, and participants who used social media reported significantly higher levels of worry compared to those who used television.

According to the Dual-Factor Model of mental health [32], people can experience high levels of well-being even in the presence of negative experiences, and vice versa. In our study, we evaluated life satisfaction using an item from the PMH-Scale and assessed the level of worries by examining the frequency of concerns regarding the impact of political developments on one’s own life or the lives of one’s family. It is well known that socio-political challenges, climate crises, and pandemics can significantly impact civilian mental health, leading to psychological impairments and decreased life satisfaction [1, 6, 14, 15]. Worries about various life domains can vary over time [37]. Political, cultural, and climate changes, along with crises, influence well-being, life satisfaction, and the level of worries [37].

### Bivariate and multivariate associations between sociodemographic variables, life satisfaction, worries, and main source of information

Based on the correlations, we found that lower life satisfaction and increased worries are significantly associated with younger age. This finding is consistent with other studies indicating lower life satisfaction in younger age groups [38–40] and higher levels of worry regarding COVID-19 infection and climate change among younger individuals compared to older ones [41–43].

At the multivariate level, we found that females report higher life satisfaction than males. The literature presents mixed results regarding the relationship between life satisfaction and gender [44]. Studies indicate that factors such as income, education, national contexts, and age influence this relationship [45–47]. Our finding that higher life satisfaction is associated with greater years of education aligns with common results in other studies [48] and may be confounded by income [49]. Additionally, worries were found to depend on both the region of residence and the type of media use. The observation of higher worries in the eastern parts of Germany may be confounded by other sociodemographic variables such as wealth and age. Even 35 years after the reunification of East and West Germany, wealth disparities still exist [50]. Several studies have documented an association between worries and age [37, 51]. In East Germany, there remains a lower socioeconomic status, and an older population compared to West Germany [50, 52].

According to the information source, this survey revealed that the most commonly used sources of information were television and the Internet. This finding is consistent with the findings of a large Europe-wide panel survey conducted by van Aelst et al. [22] that reported an increase in television news consumption and an increase in the reliance on the Internet and social media for news and information. These news sources are easily accessible and offer a more immediate coverage, thus having the potential to fulfil the need for a quick orientation in times of crisis and crisis-specific concerns [22]. However, the use of these sources of information is associated with a higher risk of receiving unbalanced information and misinformation. This is primarily driven by the use of algorithms and the non-restrictive dissemination of information [27–29].

Furthermore, the observation that lower life satisfaction and higher worries are associated with increased social media use corroborates studies showing that social media use is linked to poorer mental health, reduced well-being, and heightened anxiety [18–20, 23–26]. In a review of 43 studies, negative aspects of social media use included social isolation, harm, depressive symptoms, and a heightened risk of bullying [53]. Further studies have shown that prolonged screen time on social media platforms may mediate the association with higher mental health symptoms and poor well-being [54–57]. Social comparisons and the increased risk of social isolation following rejections on social media could also serve as mediating factors [58]. The fact that higher social media use was associated with both aspects of mental health, while sociodemographic variables were differently connected to these aspects, underscores the significant impact of social media on mental health. Against this background, it is important to critically reflect on one’s own media use to maintain adequate levels of mental health while staying informed about current world affairs. Consequently, there is a need for enhanced media literacy and targeted psychological interventions for vulnerable groups. To support this, mental health literacy programs ought to incorporate the meaning of social media use for mental health, especially in times of crisis and uncertainty.

### Limitations

Despite the novelty of our results, there are some limitations that need to be considered and should be tackled in further research. First, our results are limited by the lack of information on the income or wealth of the studied population. This gap restricts the interpretation of the associations between worries and life satisfaction with other sociodemographic variables. Wealth may be an important factor mediating the relationship between worries and region of residence, as well as the association of life satisfaction with gender and years of education [45–47, 49, 50]. In the context of worries and mental health, studies suggest that the content of worries is an important factor to consider [43, 59]. Regarding social media use, research indicates that the quantity and quality of social media usage are key factors in examining its impact on mental health outcomes [60]. Future studies should examine these factors for a more detailed understanding of the associations and a better generalizability of the findings. Second, our results are only correlational in nature, which prevents us from making definitive statements about how and in which direction mental health is influenced by sociodemographic variables and social media use in times of the current changes and crises. Prospective studies are required to track time-dependent changes in mental health [37] and to draw causal conclusions. Furthermore, we utilized single items to measure life satisfaction, worries, and media use. Employing well-established, validated questionnaires would have increased the reliability of the results. Third, the analyses were conducted for the overall sample with an age range from 14 to 99 years. Due to the non-normally distributed age groups, we did not conduct bivariate and multivariate analyses for specific age groups and only included age as a covariate in the MANCOVA. As previous studies have shown differences in the experience and consequences of crisis situations [61, 62] and in media use depending on age [63, 64], we recommend future studies to conduct separate analyses for different age groups. Lastly, this study was not pre-registered. To avoid duplication of research efforts, particularly when addressing timely topics, and to increase transparency, this should be considered in future studies.

## Conclusion

This study provides insights into life satisfaction and worries within a representative German sample, considering current environmental and socio-political changes and crises one year after the COVID-19 pandemic. The results reveal that the majority of the sample are satisfied with their lives. However, they are also concerned about various life domains. This finding supports the Dual-Factor Model and emphasizes the significant influence of factors such as gender, education, regional differences, and (social) media usage on both life satisfaction and worry levels. These insights underline the need for enhanced media literacy and targeted psychological interventions for vulnerable groups.

## Data Availability

The dataset generated and analyzed during the current study is available from the corresponding author upon reasonable request.

## Abbreviations

CATI: Computer-assisted telephone interviewing
MANCOVA: Multivariate analysis of covariance
PMH-Scale: Positive Mental Health Scale
